# Points from Hospital Reports

**Published:** 1924-03

**Authors:** 


### POINTS FROM HOSPITAL REPORTS.

### Coventry and Warwickshire Hospital.

This hospital is in the gratifying position of having closed its financial year free of debt. This is peculiarly satisfactory, since at one time the debt was as high as £42,000. At the beginning of the year it stood at £25,000, and much energy and determination were required to bring about this excellent result. In fact nothing but the united efforts of all sections of the community could have accomplished so much. A Fancy Fair brought in £7,900, a Standard Motor Ballot £1,600, a Hospital Carnival £2,912. The Warwickshire Miners' Welfare Fund gave the handsome donation of £1,000, and the number of legacies was larger than usual. Fortunately for the clearing of the debt, says the Report, the past year was the Hospital Saturday Fund's Jubilee Year, and that Committee sought to celebrate it by handing over a record collection by means of a School Children's Procession, Flower Day, Whist Drive, etc., and with the "Standard Ballot" and Hospital Carnival proceeds which were handed over to their Jubilee Fund they managed to raise the unprecedented sum of £22,500. The highest record before was £15,000. They undoubtedly take the lead of all Hospital Saturday Committees throughout the country and deserve the highest praise for their untiring efforts. The employers' scheme of contributions is well supported—indeed, the Coventry employers seem to set an example to those in other industrial centres.

### N.S.W. Hospital Saturday Fund.

This annual report of the New South Wales Hospital Saturday Fund is for the year ending May 31, 1923, and is a very concise, clear and business-like document. The operations of the fund in this, the twenty-ninth year of its existence, produced an amount somewhat smaller than in the next preceding year, the figures being £28,243 as compared with £29,725. On investigation, however, we were pleased to find, firstly, that the falling-off was not in industrial collections or subscriptions and donations, which are the real criteria of popular and working-men's support, both of which showed a substantial increase; and secondly, that it was purely adventitious, resulting from heavy rain falling just at the most productive hour of the day and practically extinguishing the out-door collections and the counter-sales. The amount distributed among some thirty-seven institutions was £25,705, the Royal Alfred Hospital, Sydney, receiving the largest amount (£3,694), and the Nepean District Hospital the smallest. The death of Sir Denison Miller, Vice-Chairman of the Board and Chairman of the Executive Committee, is a very serious loss to the fund. He was evidently a veritable pillar of the Hospital Saturday movement in New South Wales, as he was indefatigable and constant in attention to its affairs, notwithstanding the exacting demands of his official position of Governor of the Commonwealth Bank of Australia. The registered office of the fund is in Sydney.

### Preston Royal Infirmary.

The Preston and County of Lancaster Queen Victoria Royal Infirmary, called for short—and not, it will be agreed, without provocation—the Royal Infirmary, Preston, is a flourishing and progressive institution. Two hundred and forty-three more patients were treated in its 160 beds than in 1921, and the number of out-patients was also greater. The income was within £22 of equalling the total expenditure, of which £6,529 was extraordinary. It was, moreover, £2,488 in excess of that for 1921. There was a reduction of £1,500 in the ordinary expenditure. This is a succinct summary of the work and finance of the hospital for 1922, and represents a thoroughly satisfactory state of things. One of many points upon which the hospital is to be congratulated is the very substantial sum contributed by workpeople, no less than £8,644; and, what is even more satisfactory still, it is rapidly increasing year by year. The figures for 1920, 1921 and 1922 are £3,810, £6,261 and £8,644 respectively. This is remarkable progress, especially when we bear in mind the condition of trade and manufactures; and when we find that the in-patients' payments amounted to £3,606, making, with workpeople's contributions, no less than £12,250 from actual and potential patients, and observe also that the ordinary expenditure of the hospital is but £19,513, a situation is shown which can only be described as extremely satisfactory. Would that it were possible for London to evolve such a state of things! Three important extensions were opened out during the year—the Lostock Hall Convalescent Hospital for Women and Children, the Electro-Massage Department and the Maternity Ward. A beginning was also made with the urgently-needed extension of the Nurses' Home. The report of the Board of Management concludes with a recital of improvements which should be carried out in the early future if the infirmary is to be kept up to date. This report is excellently printed on very good paper, contains several admirable and interesting illustrations, and is altogether an attractive book.

Derbyshire Royal Infirmary.

The examination of this annual report, which deals with the year ended September 28, 1923, is one of the most agreeable experiences which, as reviewers of hospital reports, we have had the good fortune to meet with. As we turned over the pages and one after another the various phases of the work presented themselves—relief, expenditure, income—we found ourselves repeating the expression, "highly satisfactory." Once, indeed, we involuntarily echoed the ejaculation of the Board of Management, "wonderful"; and as we turned the last page we instantly recognised the striking appropriateness of the words with which the President of the Infirmary (the Marquess of Hartington) concludes the report of the Board of Management, *Laus Deo.* To come to details, the extent of the relief afforded, in other words, the work of the hospital, exhibits a steady, continuous, all-round increase. The expenditure, on the other hand, shows a material diminution, a decline in 1922 of £4,500, and in 1923 of £1,400, while the income has waxed rapidly and yet continuously. It is in this last connection that the most remarkable phenomenon is recorded. It is not so much that, taken over a period of ten years, the income has been developed at a greater rate, 315 per cent., than the war has augmented the expenditure, 275 per cent., which indeed is extraordinary enough, but that the Hospital Saturday organisation, which in 1914 furnished £2,921, produced in the last four years in round figures £9,700, £10,800, £12,300 and £13,500 respectively. This is approximately one-third of the sum required to meet the total ordinary expenditure of the Infirmary. It is indeed wonderful, and not less so when viewed in association with another noteworthy fact, that "donations" exhibit an advance of almost equal magnitude and steadiness. Despite an increase in expenditure unparalleled in extent, and until recently utterly beyond control, we find that whereas in 1914 the income was insufficient to meet the ordinary expenditure, in 1923 the expenditure was £2,200 within the income.

"THE HOSPITAL AND HEALTH REVIEW"

